# Amyloid precursor protein modulates β-catenin degradation

**DOI:** 10.1186/1742-2094-4-29

**Published:** 2007-12-10

**Authors:** Yuzhi Chen, Angela M Bodles

**Affiliations:** 1 Department of Geriatrics, University of Arkansas for Medical Sciences, Little Rock, AR 72205, USA; 2 Department of Neurobiology and Developmental Sciences, University of Arkansas for Medical Sciences, Little Rock, AR 72205, USA

## Abstract

**Background:**

The amyloid precursor protein (APP) is genetically associated with Alzheimer's disease (AD). Elucidating the function of APP should help understand AD pathogenesis and provide insights into therapeutic designs against this devastating neurodegenerative disease.

**Results:**

We demonstrate that APP expression in primary neurons induces β-catenin phosphorylation at Ser_33_, Ser_37_, and Thr_41 _(S33/37/T41) residues, which is a prerequisite for β-catenin ubiquitinylation and proteasomal degradation. APP-induced phosphorylation of β-catenin resulted in the reduction of total β-catenin levels, suggesting that APP expression promotes β-catenin degradation. In contrast, treatment of neurons with APP siRNAs increased total β-catenin levels and decreased β-catenin phosphorylation at residues S33/37/T41. Further, β-catenin was dramatically increased in hippocampal CA1 pyramidal cells from APP knockout animals. Acute expression of wild type APP or of familial AD APP mutants in primary neurons downregulated β-catenin in membrane and cytosolic fractions, and did not appear to affect nuclear β-catenin or β-catenin-dependent transcription. Conversely, in APP knockout CA1 pyramidal cells, accumulation of β-catenin was associated with the upregulation of cyclin D1, a downstream target of β-catenin signaling. Together, these data establish that APP downregulates β-catenin and suggest a role for APP in sustaining neuronal function by preventing cell cycle reactivation and maintaining synaptic integrity.

**Conclusion:**

We have provided strong evidence that APP modulates β-catenin degradation *in vitro *and *in vivo*. Future studies may investigate whether APP processing is necessary for β-catenin downregulation, and determine if excessive APP expression contributes to AD pathogenesis through abnormal β-catenin downregulation.

## Background

β-Catenin plays a central role in Wnt signalling in the canonical pathway [1]. In the absence of Wnt signalling, cytoplasmic β-catenin exists in a complex together with axin, adenomatous polyposis coli (APC), and glycogen synthase kinase (GSK)-3β. GSK-3β constitutively phosphorylates β-catenin at Ser_33_, Ser_37_, and Thr_41 _(S33/37/T41) residues, triggering ubiquitinylation by a Cullin-1-containing E3 ligase (also known as the SCF complex) before proteasomal degradation [2-6]. Signalling by Wnt through Frizzled and LRP cell surface receptors inhibits GSK-3β and stabilizes β-catenin. When stabilized, β-catenin translocates to the nucleus and functions as a transcription cofactor of the T cell factor (TCF), activating responsive genes such as cyclin D1 and c-myc.

Several lines of evidence suggest that APP influences β-catenin regulation. APP binds to APP-BP1 which activates the small ubiquitin-like protein Nedd8 [7,8]. Activated Nedd8 modifies Cullins which then becomes more stable. When Cullin-1 in SCF is transiently stabilized, it increases β-catenin ubiquitinylation and degradation [9]. In addition, APP plays an important role in cell-cell adhesion [10], a function that may involve β-catenin. Membrane-associated β-catenin anchors cadherins to the actin cytoskeleton [11]. Interactions with cadherins may underlie an important role for β-catenin in synaptic integrity of neurons [12]. Synaptic dysfunction is one of the earliest events in AD pathogenesis [13], and APP appears to contribute to synapse formation or stabilization [14-16].

Both APP and Presenilins are genetically associated with AD. In addition to its role in APP processing, Presenilin-1 (PS1) also interacts with the cadherin/catenin adhesion complex [17] and affects β-catenin delivery to the membrane adhesion complex [18]. There is some controversy surrounding the influence of PS1 and its FAD mutants on β-catenin [19-26]. Similarly, different levels of β-catenin have been reported in AD [27-29]. This is likely attributable to the complexity of these systems made possible by multimodal interactions between APP, PS1, β-catenin, and E-cadherin; a complexity that justifies more detailed analyses of these systems. The purpose of this report is to determine whether APP mediates β-catenin degradation *in vitro *and *in vivo*. We provide critical evidence that APP downregulates β-catenin in neurons.

## Methods

### Primary neurons

Pregnant Sprague-Dawley rats (E18, Charles River, Wilmington, MA) were euthanized after being rendered unconscious with CO_2 _according to the protocol approved by the Institutional Animal Care and Use Committee. The cortices from embryonic brains were gently triturated with a fine-tipped transfer pipette right after dissection. Single cells were counted and immediately plated in Neurobasal medium containing B27 supplements (Invitrogen, Calsbad, CA), 0.5 mM glutamine, 1% FBS, 1% equine serum, and 1× penicillin/streptomycin (Sigma, St. Louis, MO) at a density of 4 × 10^6 ^per 60-mm dish or 4 × 10^5 ^per well of 24-well plate on poly-D-lysine-coated dishes. The medium was replaced with fresh medium at 2 h after plating. Half of the medium was replaced with fresh medium every three days. The adult rat brain and other organs were dissected and frozen on dry ice before lysis and immunoblot analyses. Primary neurons were used at 7 DIV unless noted otherwise.

### HSV expression vectors and viral titers

Constructs of full length APP (APP_695_), mycAPP-BP1, and LacZ (expresses β-galactosidase) in the pHSVprPUC vector [7,30] were packaged into HSV-1 virus using the 2-2 cell line as described by Lim et al. [31]. Each viral stock infectious unit (IU) was determined by counting ten randomly selected fields of infected primary neurons immuno-stained for APP and myc tag, or cytochemically stained for β-galactosidase using X-gal substrate (Roche).

### Short hairpin RNA (shRNA)

RNA interference was achieved via shRNAs expressed from the pHSVGET vector for neuronal expression [32]. Each shRNA duplex was designed with a TCA AGA G loop. The APP shRNA was targeted to the following sequence: 5'-GCA GAA GAT GTG GGT TCA AAC-3' (APP1996). The control shRNA included the missense sequence 5'-GCT TCA TAA GGC GCA TAG CTA-3'. In some experiments, the vector pHSVGET was used as a control. All constructs were sequenced before HSV viral packaging. Viruses were packaged as described [31]. The IU of each shRNA virus was determined by the number of EGFP-expressing cells after infecting primary neurons for 15 hours. EGFP-positive cells were visualized by fluorescence microscopy after fixation in 4% paraformaldehyde for 15 min at room temperature.

### Neuron infection, western blots, and antibodies

For all western blot analyses, primary neurons in 60-mm dishes were infected at 7 DIV with 1 or 2 IU per cell (for protein expression), or at 0.5 or 1 IU per cell (for shRNA knockdown analyses). At 14–15 h after infection, cells were washed with cold phosphate-buffered saline (PBS) and sonicated in lysis buffer (150 mM NaCl, 50 mM sodium fluoride, 50 mM Tris-HCL, 10 mM β-glycerol phosphate, 5 mM EDTA, 5 mM iodoacetamide, 1 mM phenylmethylsulfonylfluoride, 1 mM sodium orthovanadate, 1% NP-40, 0.5% sodium deoxycholate, 0.1% SDS, pH 8.0). Protein concentrations were determined by the bicinchronic acid (BCA) method; equal amount of protein was boiled in SDS sample buffer and resolved on SDS-PAGE gels. The following antibodies were used: rabbit anti-β-catenin and mouse anti-γ-tubulin (both from Sigma); anti-phosphorylated β-catenin antibodies, S33/37/T41 and S45 (both from Cell Signaling); rabbit anti-APP antibody (369, a gift from Dr. Sam Gandy). Chemiluminescence reactions were carried out using ChemiGlow (Alpha Innotech) and the blot digital images were collected by the AlphaEase FC™ Imaging System and the FluorChem™ IS-8800 software (Alpha Innotech). Multiple exposures of each blot were routinely taken, and the exposure that had specific protein bands within the linear range in optical density was used for densitometry.

Quantitative western blot analyses were based on three or more independent experiments after densitometry tracing of specific protein bands using the Scion Image software (version 4.0.3.2, Scion Corporation). The optical densities of the protein bands in equal area of space were determined after subtracting the background per individual lane. In order to analyze the results across different experiments, the optical density levels were then normalized to γ-tubulin expressions in the respective western blot. The normalized density units were then analyzed by one-tail or two-tail *t*-Tests (more stringent) for two samples assuming unequal variance using the EXCEL data analysis tool (Microsoft Corporation). A *p *value of 0.05 or less was considered to be statistically significant.

### Cell membrane, cytosolic and nuclear fractions

The post-nuclear membrane fraction was isolated as described [33]. Briefly, after transfection of HEK293 cells for 40 hours, or after infection of primary neuronal cultures for 15 hours, the medium was withdrawn, and cells were harvested in ice-cold PBS, washed once, and homogenized by shearing through a 22-ga needle in buffer HS (250 mM sucrose, 150 mM NaCl, 10 mM Tris-HCl, and 5 mM EDTA, pH 7.4) supplemented with a mixture of protease inhibitors (Sigma). All subsequent steps were carried out on ice unless indicated otherwise. The crude nuclear fraction was saved after the samples were centrifuged at 1500 × *g *for 10 min and resuspended in buffer HD (10 mM Tris-HCl, pH 7.4, 150 mM NaCl, 1% SDS, and 5 mM EDTA) with protease inhibitors. Membrane fractions were collected from the postnuclear supernatants by centrifugation for 20 min at 14,000 × *g *and resuspended in buffer HD with protease inhibitors. Proteins in the supernatant (cytosolic fraction) were precipitated with 20% trichloroacetic acid. The precipitated proteins were washed with 95% ethanol. Protein pellets were resuspended in 1% Triton X-100 containing protease inhibitor mix (1:250). Equal amount of proteins from membrane, cytosolic or nuclear fractions were resolved by SDS-PAGE, transferred to nitrocellulose membrane, and analyzed for β-catenin by western blot analyses.

### Dual luciferase assays

HEK293 cells were transfected with pGL3-OT or pGL-OF (improved versions of pTOPflash and pFOPflash, respectively) [34] and APP or a mutant APP construct. Renella luciferase reporter (CMV-PRL, Promega) was included in each sample to normalize transfection efficiency. The positive control was prepared from cells transfected with β-catenin [35]. Cells were harvested at 40 h after transfection and dual luciferase assays were performed as described in the manufacturer's protocol (Promega). Luciferase activity was measured by a dual-injector microplate luminometer (Turner Biosystems) using Veritas program.

### Immunofluorescence microscopy

Primary neurons were fixed with 4% paraformaldehyde for 40 m at room temperature. Cells were then washed with PBS, blocked with 3% normal goat serum, and stained with rabbit anti-β-catenin (Sigma) for 2 h at room temperature. Cells were then washed and incubated with Alexa Fluor 488-conjugated goat anti-rabbit for 1 h (Molecular Probe). Counterstaining with DAPI was performed in some cells by incubating for 2 m with DAPI (20 μg/ml). After 4× washes in PBS, cells were mounted in 90% glycerol. Negative controls were without primary antibodies. The specificity of the anti-β-catenin antibody was also demonstrated in western blots. Confocal images were taken under a 63× objective with a Nikon D-Eclipse C1 confocal microscope through z-series at the same settings. Individual z-sections and composite images were further grouped in Photoshop (version 6.0, Adobe).

### APP knockout animals and immunofluorescence labelling

APP knockout animals were raised and genotyped as described before (n = 4; 3 were 8 months old and 1 was 18 months old) and WT controls (n = 4; 3 were 8.5 months old and one was 7 months old) [36]. Brain sections (6 μm thick) were pre-incubated with 2N HCl for 30 m, washed 6×, blocked with 3% normal goat serum, and incubated overnight in primary antibodies. The sections were then washed 3×, and incubated in secondary antibodies for 1 h before incubation in DAPI solution for 2 m. Sections were washed and mounted in 90% glycerol. Images were obtained at the same settings with a Nikon Eclipse 600 fluorescence microscope and CoolSnap digital camera using MetaVue software (version 6.2r2, Molecular Devices Corporation). The pixel density of the CA1 pyramidal cells in the cell body layer were sampled using the MetaVue software. The mean value of pixel density was calculated and compared by two-tailed *t*-Test assuming unequal variance (Excel, Microsoft).

## Results

### APP downregulates β-catenin in primary neurons

**Figure 1:**
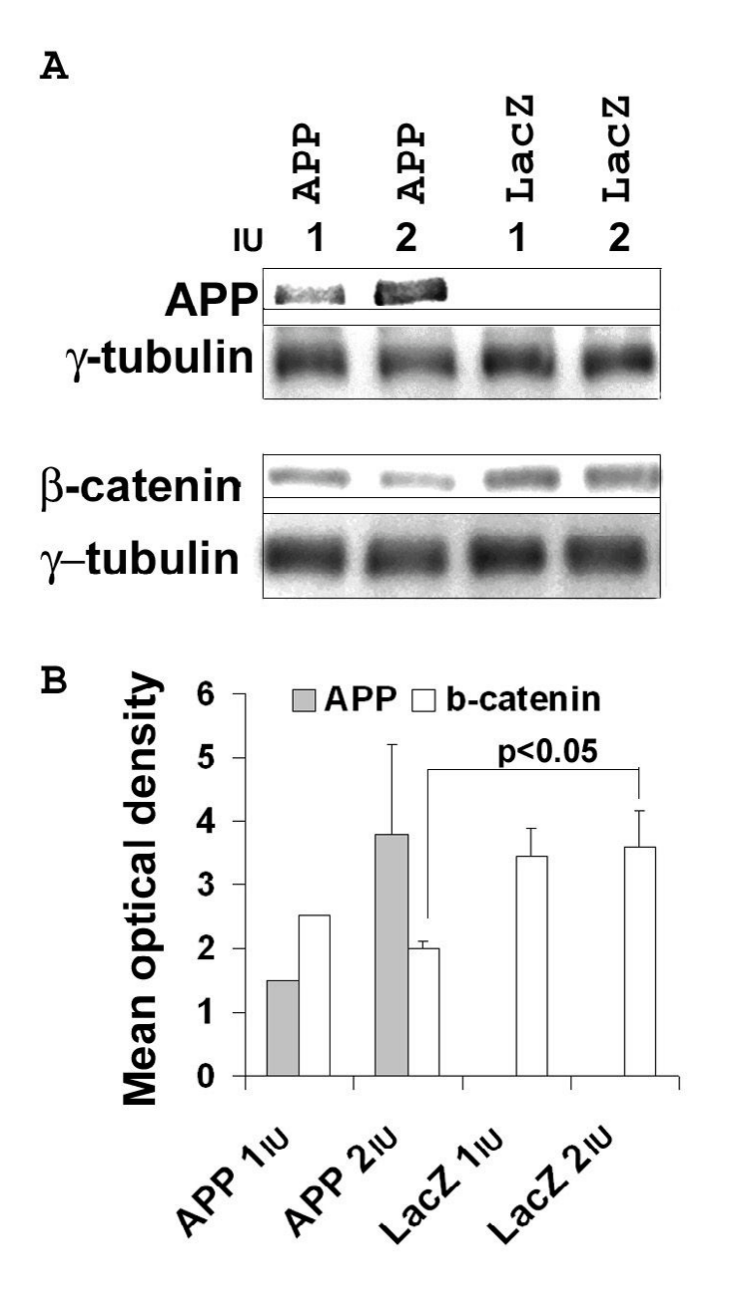
**APP expression induced degradation of β-catenin**. A. Overexpression of APP reduces β-catenin levels. Human APP_695 _or the control LacZ was expressed in primary neurons via HSV vectors at 1 or 2 IU per cell during 14 h of infection. The control HSV vector carries the β-galactosidase gene (LacZ). B. Quantitative densitometric analyses of western blots from 4 independent experiments. An average 44% reduction in normalized mean optical density (representing protein levels) was observed for 2 IU/cell of APP compared to the corresponding LacZ control (*p *< 0.05, *t*-Test). Shorter exposures were used for densitometry analyses; longer exposures also showed the expression of endogenous APP, but densitometric analyses were not possible due to the solidified chemiluminescence reaction products.

To determine the effect of APP on β-catenin, full length human APP_695 _was expressed in primary neurons using HSV-1 vector-mediated gene transfer. An increase in APP levels resulted in a significant decrease in total β-catenin (Fig. 1). Quantification indicated that a tripling of APP (at 2 IU of virus) was associated with a reduction of β-catenin to approximately half of that in the control (LacZ).

### APP facilitates β-catenin degradation by increasing β-catenin phosphorylation at S33/37/T41

**Figure 2:**
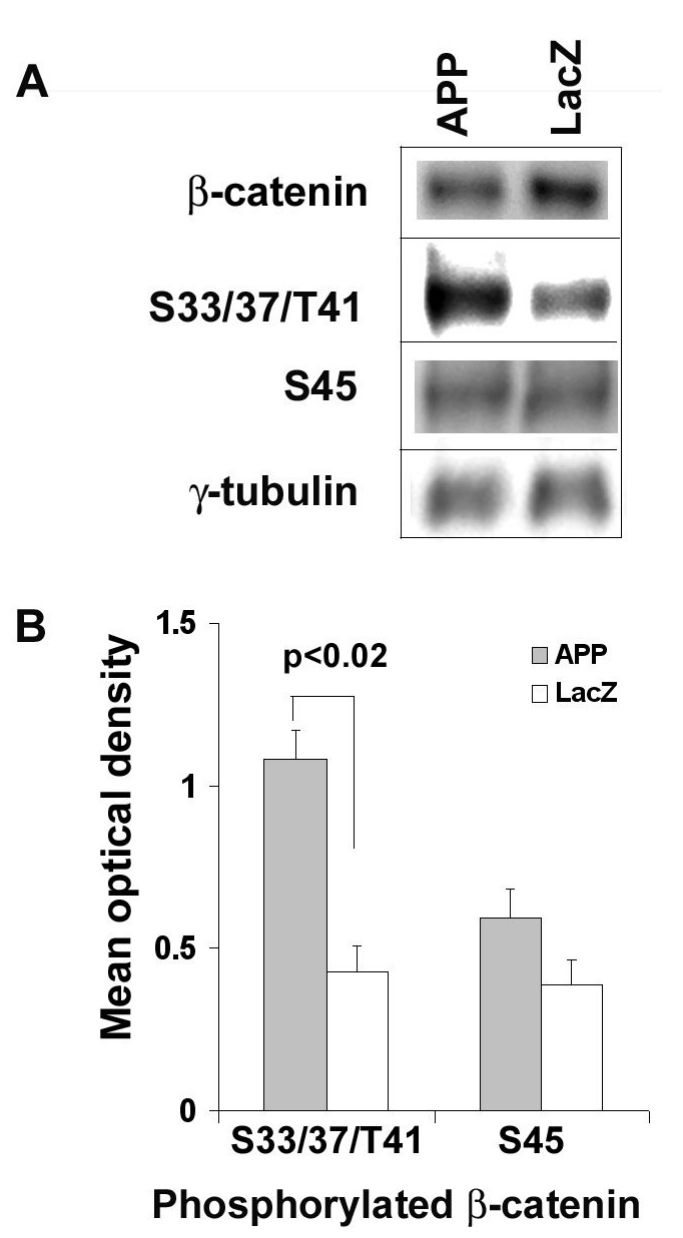
**APP facilitates β-catenin degradation by increasing β-catenin phosphorylation at S33/37/T41 residues**. A. Primary neurons infected with APP contained less steady-state β-catenin and higher levels of phosphorylated S33/37/T41 than did neurons infected with control LacZ (2 IU virus per cell). Levels of phosphorylated S45 did not appear to change. B. Quantitative analyses of western blots from 3 independent experiments. APP significantly increased β-catenin phosphorylation at S33/37/T41 residues (p = 0.02, *t*-Test), but not at S45 residue (p = 0.19, *t*-Test).

Levels of β-catenin are tightly regulated by phosphorylation, ubiquitinylation, and proteasomal degradation. Amino acids S33/37/T41 of β-catenin are co-ordinately phosphorylated by GSK-3β before ubiquitinylation and degradation. Casein kinase 1 additionally phosphorylates β-catenin at Ser_45 _(S45) [37]. To determine whether APP affected these phosphorylation events, we used β-catenin phosphorylation-specific antibodies to compare neurons infected with APP or the control virus (LacZ). Statistical analyses revealed that APP over-expression in primary neurons significantly increased S33/37/T41 phosphorylation (*p *< 0.02) without significantly altering S45 phosphorylation. Representative blots along with quantitative analyses are presented in Figure 2.

### Downregulating endogenous APP significantly inhibits β-catenin phosphorylation at S33/37/T41 but not at S45

**Figure 3:**
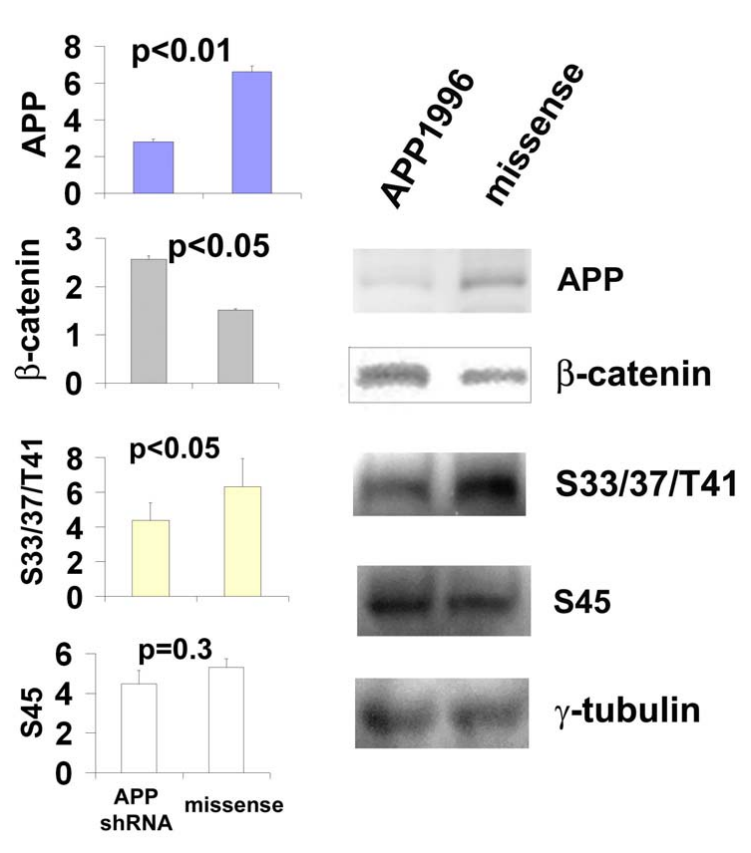
**Suppression of endogenous APP results in increased total β-catenin and decreased S33/37/T41-phosphorylated β-catenin**. Primary neurons were infected with APPshRNA (APP1996) or missense shRNA viruses (0.5 IU/cell) for 14 h before harvest and protein analyses. The mean optical densities of 3 independent experiments for each protein analyzed are presented in the Y-axis in the bar graphs. The statistical significance of difference is also presented in each bar graph (*p *values from *t*-Test) for each protein analyzed (labelled on the Y-axis). Representative blots are shown on the right. Loading controls were γ-tubulin reprobed from the same blots.

To exclude the possibility that enhanced β-catenin degradation was due to a gain-of-function artifact when APP is over-expressed, we used siRNAs to suppress the expression of endogenous APP. An HSV-1 shRNA vector was used to express siRNAs (APP1996) targeting rat APP in primary neurons; no human APP was expressed in these experiments. Protein levels were quantified by densitometry, with tracing and normalization to the levels of γ-tubulin on each blot. Statistical analyses by *t*-Test revealed that suppressing the endogenous APP by this means resulted in a significant increase in total β-catenin compared to the control (missense shRNA) (Fig. 3). APP downregulation also significantly reduced β-catenin phosphorylation at S33/37/T41, but did not significantly affect phosphorylation at S45. These results extend the inverse relationship between APP and β-catenin levels.

### APP-expression decreases β-catenin in membrane and cytosolic fractions without affecting nuclear β-catenin

**Figure 4:**
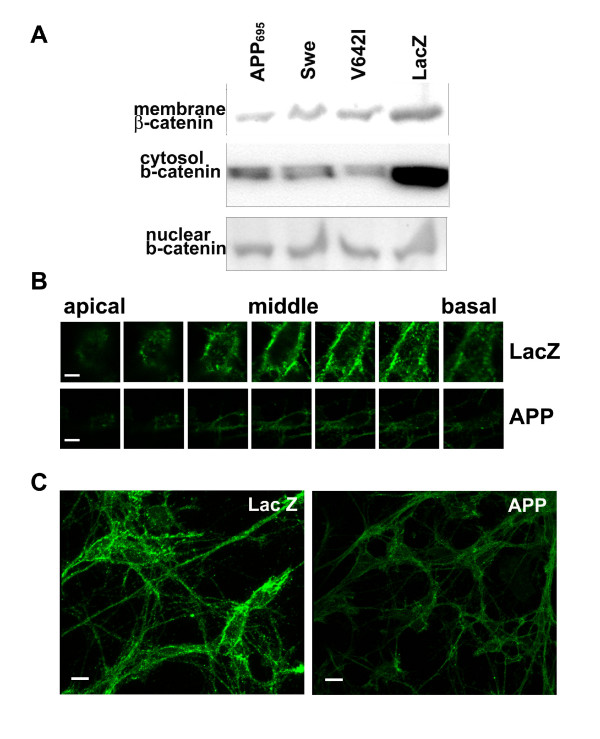
**APP expression downregulates β-catenin levels in membrane and cytosolic fractions but not in the nuclear fraction**. A. β-Catenin levels from membrane and cytosolic fractions isolated from primary neurons. Wild type or FAD APPs were overexpressed in primary neurons via HSV-mediated gene transfer using LacZ as the control. 10 μg protein per sample was analyzed in each fraction. B. Selected z-series of single cells. Scale bar, 2 μm. C. Composite images from z-series. Scale bar, 5 μm. In both B and C, primary neurons were infected with APP or the control LacZ viruses at 2 IU/cell for 14 h. Cells were then fixed and stained with rabbit anti-β-catenin and Alexa fluor 488 goat anti-rabbit. Images were collected at the same settings for fluorescence intensity comparison.

The function of β-catenin differs depending on its cellular location. We sought to determine if APP expression differentially affects β-catenin levels in crude preparations of membrane, cytosolic or nuclear fractions. Three forms of APP were tested: wild-type, Swedish mutation (Swe), and London mutation (V642I). In primary neurons, wild-type APP and each of the FAD mutants dramatically reduced total β-catenin compared to the control (LacZ) in both membrane and cytosolic fractions (Fig. 4A). APP decreased β-catenin levels to 48% of control in the membrane and to 29% of control in the cytosol (average of two independent experiments in primary neurons). These results were corroborated by data from HEK293 cells transfected with the same APP cDNAs expressed from pcDNA3 constructs (data not shown).

In contrast to membrane and cytosolic β-catenin, nuclear β-catenin did not appear to change upon expression of wild-type or FAD APP (Fig. 4A). To obtain further support for the fractionation experiments, we then tested the nuclear function of β-catenin using luciferase reporter assays. HEK293 cells were transfected with wild-type or FAD APP, along with the TCF luciferase reporter plasmid (pGL3-OT). None of the APP constructs consistently decreased pGL3-OT luciferase reporter activity. Controls included cotransfection of a mutant TCF luciferase reporter (pGL3-OF) with APP, and cotransfection of pGL3-OT with pcDNA3 (data not shown). A positive control was prepared from cells transfected with a β-catenin expression construct and the pGL3-OT plasmid. Together, these data suggest that APP may not affect β-catenin transcriptional activity.

To complement the above experiments, confocal microscopy was used to assess β-catenin levels and cellular locations in primary neurons. Primary neurons expressing APP or LacZ (control) were fixed and labelled with the rabbit anti-β-catenin antibody and the Alexa fluor 488 goat anti-rabbit antibody. Representative z-sections of the same cells are presented in Fig. 4B. The z-sections show abundant β-catenin present near or at the plasma membrane. More β-catenin seemed to localize in the basal-lateral than apical regions, and APP expression did not seem to affect β-catenin localization compared to the LacZ control. The z-series (Fig. 4B) and the composite images (Fig. 4C) also revealed that β-catenin fluorescence intensity decreased dramatically in APP-expressing neurons compared to the LacZ control, further supporting the data obtained by fractionation assays.

### β-Catenin is significantly increased in CA1 pyramidal cells from APP knockout animals

**Figure 5:**
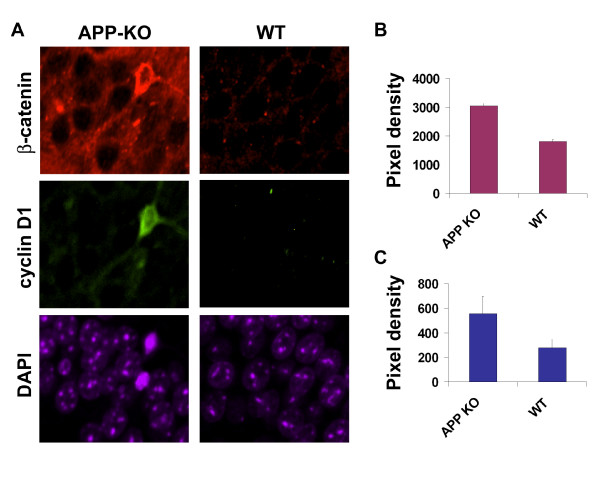
**β-Catenin and cyclin D1 are significantly elevated in hippocampal CA1 pyramidal cells in APP knockout mice compared to those in WT controls**. Brain sections from APP knockout and WT mice (n = 4 each) were co-labelled with rabbit anti-β-catenin and mouse anti-cyclin D1 together with Alexa Fluor 594 and Alexa Fluor 488 secondary antibodies. The sections were also counter-stained with DAPI to visualize the cell body layer. Images were captured at the same settings with a Nikon Eclipse 600 microscope. Representative images from an APP knockout and a WT animal are presented in A. The pixel density of specific staining was randomly sampled in the CA1 cell body layer and the mean values from each group of animals are shown in the bar graphs: B. β-catenin, two-tail t-Test, APP knockout vs AT, p = 0.001; C. cyclin D1, two-tail t-Test, APP knockout vs WT, p = 0.02.

The results presented above show that APP mediates β-catenin degradation in primary neurons. We next sought to determine if β-catenin levels are altered in CA1 pyramidal cells from APP knockout animals. Fig. 5 shows that β-catenin is significantly elevated in APP knockout pyramidal cells compared to wild-type controls (Fig. 5A &5B). These increases in β-catenin were associated with significant increases in cyclin D1 expression (Fig. 5A &5C). These data suggest that chronically increased β-catenin levels in these neurons might result in cell cycle reactivation. These data further support our findings in primary neurons suggesting that APP mediates β-catenin homeostasis.

## Discussion

In this report, we provide strong evidence that APP mediates β-catenin downregulation. We demonstrate that APP downregulates β-catenin levels in primary neurons by facilitating specific phosphorylation of S33/37/T41 residues in β-catenin. APP expression is inversely correlated with the levels of total β-catenin. Corroborating data were obtained using APP siRNAs: suppression of APP by siRNAs increased steady-state β-catenin levels and reduced the levels of S33/37/T41 phosphorylation in neurons. We further show that β-catenin accumulates in hippocampal pyramidal cells in APP knockout animals. Together, these data establish that APP is physiologically important in maintaining β-catenin homeostasis, which is likely critical for neuronal survival and function by preventing aberrant cell cycle reaction.

We show that familial AD mutants of APP induce similar β-catenin degradation, suggesting that familial AD mutants retain similar functions. Our method might not be sensitive enough to detect the degree of differences in β-catenin downregulation by these APP mutants upon acute expression in cell cultures. Long-term effects of APP mutants on β-catenin expression could be analyzed in transgenic animals. In primary neurons, APP-induced β-catenin downregulation did not appear to affect nuclear β-catenin or its transcriptional activity. However, β-catenin accumulation in CA1 pyramidal neurons from APP knockout animals does appear to affect transcriptional activity, judging from the increases found in cyclin D1 protein levels in these same neurons. These observations illustrate the limitations of luciferase reporter assays in primary neurons; they also suggest that basal levels of β-catenin are necessary for neuronal function, but that these do not necessarily affect β-catenin-mediated transcription.

Several questions remain to be answered, such as whether APP processing products, such as secreted APP, or Aβ, mediate the effect of APP on β-catenin degradation. The effect of APP on β-catenin phosphorylation could also reflect the interaction between APP and APP-BP1. APP-BP1 activates neddylation and increases ubiquitinylation by the Cullin-based E3 ligase. Ubiquitinylation of β-catenin may be facilitated by the interaction between APP and APP-BP1, which is not unidirectional as indicated by the studies in Drosophila [38]. We have recently shown that one function of APP-BP1 is to downregulate Aβ_1–42 _in primary neurons [32]. Aβ_1–42 _also appears to downregulate β-catenin (YC unpublished observations). However, it remains to be determined whether APP and Aβ_1–42 _modulate β-catenin degradation through the same pathways.

The roles of APP and PS1 on β-catenin phosphorylation may be independent of each other. We show that APP facilitates β-catenin phosphorylation at S33/37/T41 residues. Others have shown that PS1 expression can enhance phosphorylation of β-catenin at S45, which may prime the phosphorylation of S33/37/T41 [39] and facilitate membrane β-catenin delivery and degradation [17,18]. The ultimate effect of PS1 on β-catenin degradation remains controversial. Some studies have shown destabilization of β-catenin by wild-type PS1 but inactivity of the FAD-mutant forms of PS1 [20,22]; others have shown the opposite [27]. Serban et al. [24] argued that the ultimate outcome rests on the inherent levels of E-cadherin. Our new findings suggest that PS1 and APP may impinge on different components in the β-catenin degradation pathway, but the underlying mechanism needs to be further investigated.

Our new findings not only support a critical role for APP in maintaining neuronal differentiation but also support a role for APP in synaptic dynamics due to the multifaceted functions of β-catenin [40,41]. Cell cycle events are widely documented in post-mortem AD brain tissues [42]. In several AD cases which we have examined, almost all hippocampal neurons show signs of cell cycle entry as indicated by the cytoplasmic translocation of Nedd8 [7]. This cell cycle deregulation and synaptic dysfunction may well be related [43]. Our new findings suggest a dysfunction of APP in maintaining neuronal differentiation in AD neurons. However, levels of APP itself must be regulated, as high levels of APP could induce neuronal death [7,44]; our new evidence suggest that low levels of APP may be equally detrimental to neurons due to β-catenin accumulation. We observed a large amount of β-catenin in the plasma membranes of primary neurons (Fig. 4), in accord with findings reported in Drosophila [18]. Membrane β-catenin is known to play a role in dendritic remodeling in mature neurons [45]. Synaptic dynamics may involve changes in the interaction of β-catenin with cytosolic or membrane proteins such as with E-cadherin. These considerations, taken together, implicate APP as a critical factor in neuronal function that warrants further investigation.

## Conclusion

In summary, this report documents a function of APP in β-catenin downregulation *in vivo *and *in vitro*. These findings open new avenues for research involving the role of APP processing in β-catenin downregulation. Future studies may also determine whether excessive APP expression contributes to AD pathogenesis involving abnormal β-catenin phosphorylation and degradation in transgenic animals.

## List of abbreviations

APP, amyloid precursor protein; 

AD, Alzheimer's disease; 

Aβ, beta-amyloid; 

DIV, day *in vitro*; 

siRNA, small interfering RNA; 

shRNA, small hairpin RNA.

## Competing interests

The author(s) declare that they have no competing interests.

## Authors' contributions

YC conceptualized and designed all the experiments and prepared this manuscript. Both YC and AMB collected data described in this manuscript.
